# Case report: paroxysmal cold hemoglobinuria presenting during pregnancy

**DOI:** 10.1186/s12878-015-0023-7

**Published:** 2015-02-13

**Authors:** Andrea O Akpoguma, Thomas L Carlisle, Steven R Lentz

**Affiliations:** Department of Internal Medicine, University of Iowa Carver College of Medicine, Iowa City, IA USA

**Keywords:** Donath-Landsteiner antibody, Paroxysmal cold hemoglobinuria, Pregnancy, Hemolytic anemia

## Abstract

**Background:**

Paroxysmal cold hemoglobinuria is caused by a biphasic IgG autoantibody that triggers complement-mediated intravascular hemolysis. Paroxysmal cold hemoglobinuria has not previously been reported to occur in association with pregnancy.

**Case presentation:**

We report a case of an 18 year old female who presented in early pregnancy with acute hemolytic anemia and a positive Donath-Landsteiner antibody test. She was diagnosed with paroxysmal cold hemoglobinuria and treated supportively. Her hemolysis resolved within 6 weeks. Because maternal IgG autoantibodies can cross the placenta, the patient was monitored closely throughout her pregnancy for recurrence. The outcome of the pregnancy was successful, with no evidence of neonatal anemia or hemolysis.

**Conclusion:**

This patient had a classic presentation of paroxysmal cold hemoglobinuria with rapid onset of hemolytic anemia that resolved spontaneously. To our knowledge, this is the first report of paroxysmal cold hemoglobinuria presenting during pregnancy.

## Background

Paroxysmal cold hemoglobinuria (PCH) is a rare form of autoimmune hemolytic anemia mediated by a biphasic IgG autoantibody that triggers complement-mediated intravascular hemolysis. The name PCH is derived from its classic presentation with episodic hemoglobinuria, typically following exposure to cold temperature. First described by Donath and Landsteiner 1904 [1-4], PCH was once considered to be a chronic condition in adults due to its association with syphilis. Today, most cases of PCH are acute, self-limited disorders that occur following transient viral or bacterial infections in children or adults [1-5]. Post-infectious PCH is usually caused by a polyclonal IgG autoantibody with specificity for the erythrocyte P antigen [1-3,5-11]. PCH also can present in association with lymphoproliferative disorders, in which case the pathogenic autoantibody tends to be a monoclonal IgG [9,10]. To our knowledge, PCH associated with pregnancy has not been described previously. Herein, we report a case of a young, healthy female who presented during early pregnancy with acute intravascular hemolysis due to PCH.

## Case presentation

**Figure 1:**
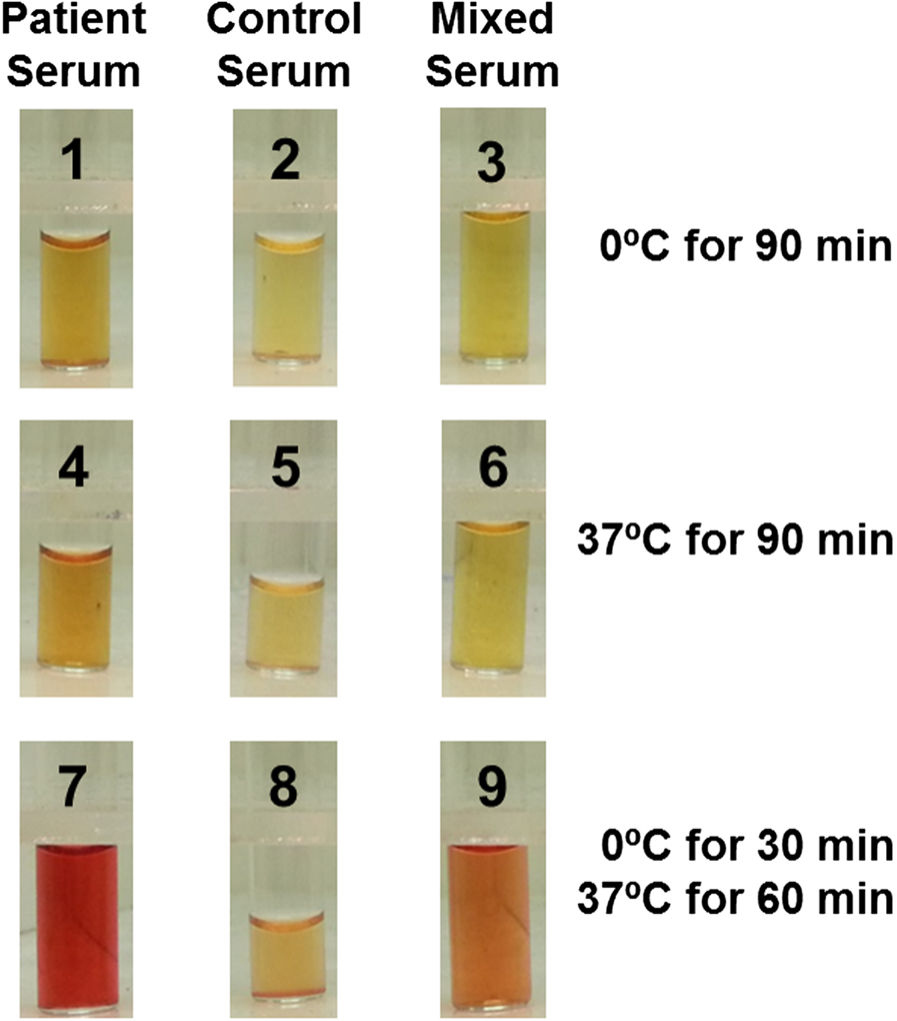
**Donath-Landsteiner antibody test.** Hemolysis of donor erythrocytes was observed when patient serum (tube 7) or a mixture of patient serum and normal serum (to provide complement; tube 9) was incubated at 0°C for 30 minutes, followed by 37°C for 60 minutes. Little or no hemolysis was seen when patient serum or mixed serum was incubated at 0°C for 90 minutes (tubes 1 and 3) or 37°C for 90 minutes (tubes 4 and 6). No hemolysis was observed in control tubes containing normal serum only (tubes 2, 5, 8).

**Figure 2:**
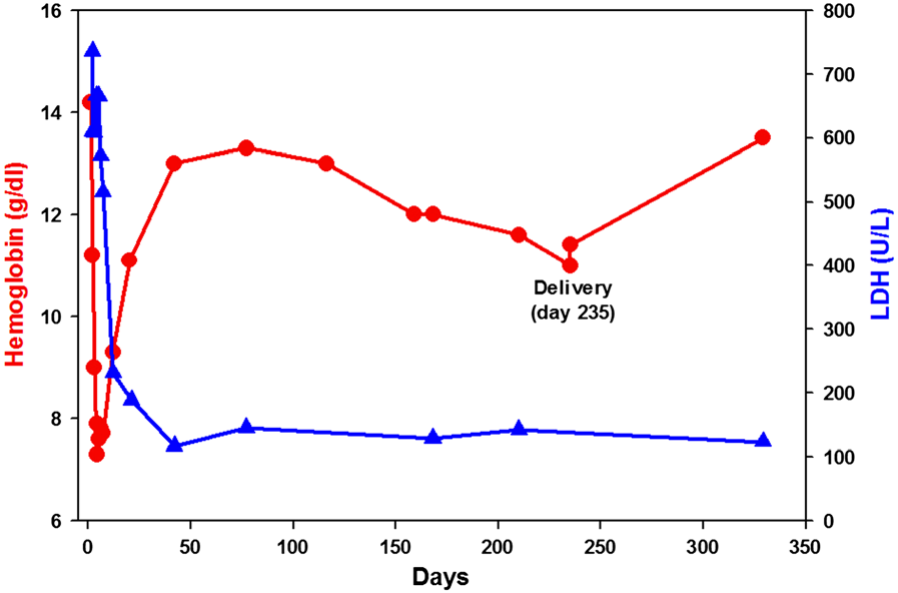
**Time course of hemoglobin and lactic acid dehydrogenase (LDH) values.**

An 18 year old female patient presented with acute symptoms of abdominal and flank pain, nausea, dark red urine, fevers and chills. One month prior to presentation, she had experienced a few days of rhinorrhea and headaches, suggestive of a viral upper respiratory tract infection. Upon presentation, she was found to be pregnant by serum hCG and ultrasound, with an estimated gestational age of 6 weeks. Laboratory evaluation showed hemoglobin 11.2 g/dL, haptoglobin <20 mg/dL, total bilirubin 6.8 mg/dL, direct bilirubin 0.7 mg/dL, lactic acid dehydrogenase (LDH) 735 U/L, and absolute reticulocyte count 49.3 K/μL. Urine analysis demonstrated 3+ blood and the microscopic examination was negative for red blood cells. A direct antiglobulin test was positive for anti-C3d and negative for IgG. The cold agglutinin titer was negative (<2). Donath Landsteiner antibody testing was performed using a blood sample that was immediately immersed into an insulated container filled with water at 37°C, and directly delivered to the testing laboratory. A Donath Landsteiner antibody test was positive (Figure 1), confirming the diagnosis of PCH. Over the next 48 hours, the patient’s hemoglobin declined to 7.3 g/dL (Figure 2). She was managed supportively with a prenatal vitamin supplement and encouraged to keep warm. Her hemoglobin and LDH normalized by day 42 (Figure 2). A Donath Landsteiner test on day 77 was negative. Her hemoglobin declined during the third trimester of pregnancy, to 11.0 g/dL on the day of delivery, without any laboratory evidence of recurrent hemolysis. She delivered a healthy female child by Cesarian section at 39 weeks of gestation. The neonate did not have anemia or hemolysis after birth.

## Conclusions

PCH was initially described in the late 1800s as a syphilis-associated chronic relapsing condition triggered by cold exposure [1-5]. Today, most cases of PCH are characterized by acute episodic hemolytic anemia that is transient and self-limited, although some cases of chronic or recurrent non-syphilitic PCH have been reported [1-3,5,11,12]. PCH is more common in children than adults, and often presents 1-2 weeks after a respiratory tract infection [1,3,5-8,11]. Severe and rapidly progressive anemia occurs, often (as in the case reported here) with a relative reticulocytopenia. The reticulocytopenia is thought to represent an ineffective bone marrow response either due to marrow suppression from viral infection or other causes [5,6,10]. Although acute episodes of hemolysis are frequently severe, PCH typically resolves spontaneously within a few days to weeks after onset. Treatment is generally supportive and consists of blood transfusions, intravenous fluids, and warming. Therapy is directed towards treating symptoms of anemia and preventing end organ complications from intravascular hemolysis. Treatment with corticosteroids and rituximab has been reported, but the value of these therapies is uncertain due to the usually transient nature of the hemolysis.

The pathogenic Donath Landsteiner autoantibody was first described in 1904 as a biphasic hemolysin that binds to red blood cells only at low temperatures and, upon warming, induces compliment activation and lysis [2,3]. In contrast to autoimmune hemolytic anemia mediated by IgM cold agglutinins, most cases of PCH are caused by non-agglutinating IgG antibodies with anti-P specificity [2,3,8]. The Donath Landsteiner antibody usually appears 1 week after the onset of illness and can persist from 1 to 3 months [8]. To avoid pre-analytic error in Donath Landsteiner testing, it is important to maintain the blood sample at 37°C until serum is separated from cells in the laboratory. This prevents adsorption of anti-P antibodies onto autologous red blood cells at low temperatures. Donath Landsteiner testing requires coordination between the clinical and laboratory teams to ensure appropriate sample collection, delivery, and testing.

The patient reported here presented in early pregnancy with acute hemolytic anemia, a negative cold agglutinin titer, and a positive Donath Landsteiner antibody test. We are not aware of a reported association between pregnancy and PCH, but other forms of autoimmune hemolytic anemia have been reported to occur more frequently among pregnant than non-pregnant women [13,14]. It is possible that the development of the Donath Landsteiner antibody in this patient was unrelated to her pregnancy, especially since she reported symptoms of an upper respiratory infection one month prior to presentation. The patient had a classic presentation with rapid onset of hemolytic anemia that resolved spontaneously. Because maternal IgG autoantibodies can cross the placenta, the patient was monitored closely throughout her pregnancy for recurrence of PCH. Donath-Landsteiner antibody testing became negative at 3 months and her hemoglobin improved to near baseline levels. She underwent an uncomplicated surgical delivery of a healthy infant who did not have any evidence of anemia or hemolysis.

### Consent

Written informed consent was obtained from the patient for publication of this case report and any accompanying images. A copy of the written consent is available for review by the Editor of this journal.

## Abbreviations

PCH: Paroxysmal cold hemoglobinuria
LDH: Lactic acid dehydrogenase
